# The Role of Sympathetic System as a Therapeutic Option in the Ischemia/Reperfusion Injury

**DOI:** 10.5935/abc.20190183

**Published:** 2019-09

**Authors:** Bertha F. Polegato, Leonardo A. M. Zornoff

**Affiliations:** 1 Universidade Estadual Paulista (Unesp) - Faculdade de Medicina de Botucatu - Departamento de Clínica Médica, Botucatu, SP - Brazil

**Keywords:** Stress, Mechanical, Simpathetic Nervous System, Hypothalamo-Hpophyseal System, Ischemia, Sympathectomy

Coronary artery disease is the leading cause of mortality and the most resource-consuming health pathology in industrialized countries.^1^ In the United States, it is believed that more than 12 million individuals have ischemic heart disease.^2^

It is estimated that approximately 1 million cases of acute coronary syndrome occur annually in the United States, demonstrating that this syndrome occurs in epidemic proportions.^2^ Few pathologies have evolved as radically as AMI, with a marked reduction in mortality as a result of changes in treatment over the past 30 years, particularly cardiac reperfusion.^1,3^

In fact, with the introduction of myocardial reperfusion, 30-day mortality has been reduced from about 14% to about 3% in several clinical trials.^3^ In addition to this benefit, early and sustained reperfusion results in lower cardiac morbidity, lower incidence of ventricular fibrillation and tachycardia, conduction disorders and less development of congestive heart failure.^1^

Despite the unequivocal benefit, an undesirable event of this strategy is the phenomenon of reperfusion injury. This phenomenon is defined as the injury that occurs as a direct result of coronary blood flow restoration. This phenomenon may have important clinical implications, because it may be responsible for 30-50% of the final infarct size.^4^ Thus, several strategies have been studied with the objective of attenuating the ischemia/reperfusion (I/R) injury phenomenon.

In this issue of the Arquivos Brasileiros de Cardiologia, Imani et al.^5^ assessed the cardioprotective effects of acute physical stress against I/R injury, through the activation of the sympathetic nervous system. They used the isolated heart preparation, with the Langendorff apparatus. The hearts were subjected to 30 minutes of ischemia, followed by 120 minutes of reperfusion. Physical stress prior to the I/R improved left ventricular developed pressure and reduced infarct size when compared with the I/R alone.^5^ In addition, chemical sympathectomy before physical stress eliminated the protective effect of physical stress on I/R-induced cardiac damages. The authors concluded that the presence of the sympathetic nervous system is necessary for the beneficial effects of acute physical stress on I/R injury.^5^

It is important to emphasize that knowledge about the pathophysiological mechanisms involved in I/R injury is critical, as this allows the creation of therapeutic strategies to attenuate or prevent cardiac damage. On the other hand, we must consider that cardioprotection strategies in I/R models are the main model used to exemplify the difficulties of translational medicine, since positive results from experimental studies are obfuscated by the fact that to date, cardioprotection strategies in clinical studies have shown negative results.^6^

Therefore, although provocative, the role of the sympathetic system as a therapeutic option in the I/R injury remains to be confirmed in future studies.
